# Effects of intraspecific competition and body mass on diet specialization in a mammalian scavenger

**DOI:** 10.1002/ece3.8338

**Published:** 2022-01-11

**Authors:** Anna C. Lewis, Channing Hughes, Tracey L. Rogers

**Affiliations:** ^1^ Evolution and Ecology Research Centre, School of Biological, Earth and Environmental Sciences University of New South Wales Sydney Australia; ^2^ The Carnivore Conservancy Ulverstone Tasmania Australia; ^3^ School of Life and Environmental Sciences The University of Sydney Sydney New South Wales Australia; ^4^ Centre for Marine Science and Innovation, School of Biological, Earth and Environmental Sciences University of New South Wales Sydney Australia

**Keywords:** body mass influence, competition, diet, foraging, scavenger, scavenging theory, specialization, stable isotopes

## Abstract

Animals that rely extensively on scavenging rather than hunting must exploit resources that are inherently patchy, dangerous, or subject to competition. Though it may be expected that scavengers should therefore form opportunistic feeding habits in order to survive, a broad species diet may mask specialization occurring at an individual level. To test this, we used stable isotope analysis to analyze the degree of specialization in the diet of the Tasmanian devil, one of few mammalian species to develop adaptations for scavenging. We found that the majority of individuals were dietary specialists, indicating that they fed within a narrow trophic niche despite their varied diet as a species. Even in competitive populations, only small individuals could be classified as true trophic generalists; larger animals in those populations were trophic specialists. In populations with reduced levels of competition, all individuals were capable of being trophic specialists. Heavier individuals showed a greater degree of trophic specialization, suggesting either that mass is an important driver of diet choice or that trophic specialization is an efficient foraging strategy allowing greater mass gain. Devils may be unique among scavenging mammals in the extent to which they can specialize their diets, having been released from the competitive pressure of larger carnivores.

## Graphical abstract

We used stable isotope analysis to analyze the degree of specialization in the diet of the Tasmanian devil, one of few mammalian species to develop adaptations for scavenging. We found that the majority of devils were dietary specialists and that only small individuals in competitive populations were true generalists. Devils may be unique among scavenging mammals in the extent to which they can specialize their diets, having been released from the competitive pressure of larger carnivores.

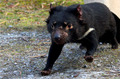

## 1 INTRODUCTION

Dietary specialization is a known predictor of population decline, leading certain species to become more vulnerable to threat of extinction (Bommarco et al., 2010; Boyles & Storm, 2007; Buechley & Şekercioğlu, 2016; Olden et al., 2008). Obligate scavengers are particularly at risk, as they must efficiently exploit resources that are inherently patchy (DeVault et al., 2003; Houston, 1979; Ruxton & Houston, 2004), toxic (Janzen, 1977), or subject to potentially dangerous competition (Buechley & Şekercioğlu, 2016; Kendall, 2012; Kruuk, 1967; Petrides, 1959; Trinkel & Kastberger, 2005; Wallace & Temple, 1987). Due to these challenges, most vertebrate scavenger species are facultative scavengers, capable of acquiring food through both scavenging and predation, while obligate scavenging is rare and thought to be exclusively displayed by large soaring birds (i.e., vultures; Ruxton & Houston, 2004). Many facultative scavenger species are common and successful, suggesting that a generalist, and therefore highly adaptable, strategy of feeding should be beneficial (DeVault et al., 2003; Wilson & Wolkovich, 2011). If most scavengers favor an opportunistic diet that combines hunting and scavenging, then it might be assumed they also would favor wide over narrow dietary niches. That is, a scavenger cannot afford to be overly selective about the food items it consumes. Despite this, some facultative scavengers show specialization in response to seasonal change and distribution patterns (Anderson et al., 2008; Masello et al., 2013), or when a greater variety of resources is available (Larson et al., 2020).

Many species show variation in resource use between individuals within a population, even among those that share similar environments, life histories, or physical traits (Bolnick et al., 2003; Van Valen, 1965; Werner & Sherry, 1987). Where this variation is high, different individuals can respond to and alter their environment in diverse ways (Araújo et al., 2011; Bolnick et al., 2011; Hughes et al., 2008). However, when characterizing the diet of an entire species, this individual variation is often ignored in order to streamline inclusion into interspecific ecological modelling, running the risk of over‐simplifying systems and misunderstanding how individual animals interact with one another (Bolnick et al., 2003). Thus, although it is often assumed that scavenging species are dietary generalists, with all individuals exploiting a wide dietary breadth by necessity, it is possible that individual specialization among scavengers is more common than previously thought.

**FIGURE 1:**
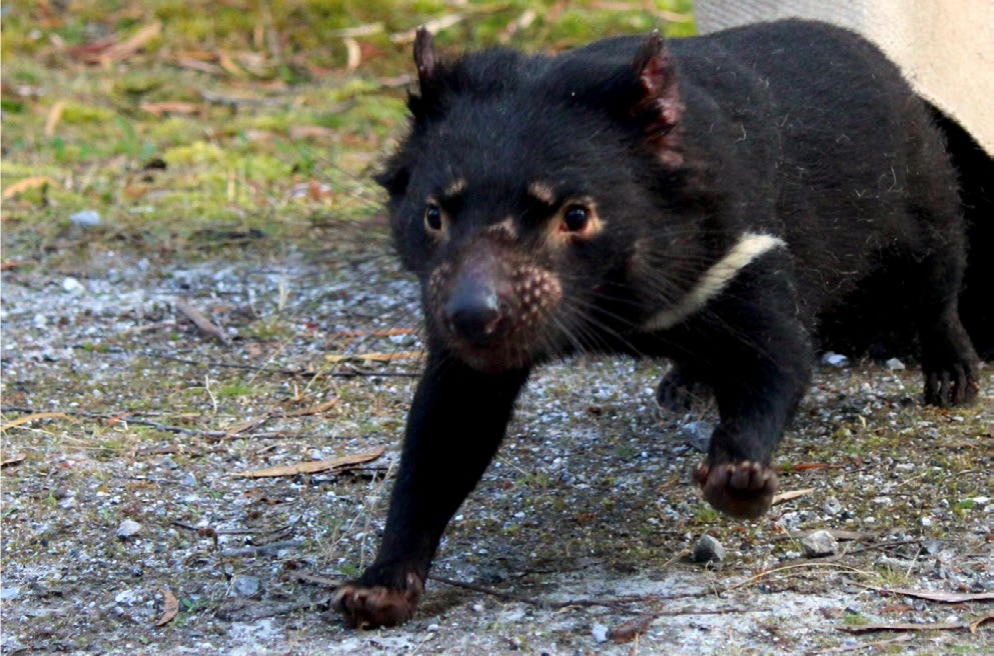
The Tasmanian devil (*Sarcophilus harrisii*), one of few mammalian species to develop adaptations for scavenging (Photo by A. Ananda)

The Tasmanian devil (*Sarcophilus harrisii*; Figure 1) is one of the few mammalian species to have developed physiological and behavioral specializations for scavenging (Brown, 2006). Some of these specializations contribute to minimizing energetic costs, including the possession of a very low basal metabolic rate (Nicol & Maskrey, 1980), an energy‐efficient gait (Brown, 2006; Guiler, 1970), and a preference for using roads for rapid movement through the landscape (Andersen et al., 2017a; Guiler, 1970). Others are more specifically designed for finding and processing carcasses: a large olfactory bulb (Patzke et al., 2014); bone‐crushing jaws (Attard et al., 2011; Wroe et al., 2005); and a propensity for gorge feeding (Pemberton & Renouf, 1993). These traits position the devil as a species heavily reliant on carrion (Jones & Barmuta, 1998; Kane et al., 2016), an assumption recently confirmed when individuals bearing video collars were recorded scavenging at 98% of feeding events (Andersen et al., 2020). The development of these traits may even conflict directly with the maintenance of traits favoring predatory behavior, as observed in avian scavengers (Houston, 1979). Yet until recently (Andersen et al., 2020; Bell et al., 2020; Cunningham et al., 2018; O’Bryan et al., 2019), the Tasmanian devil has largely been omitted from scavenger theory, more frequently referred to as an apex predator than an apex scavenger.

We propose that the devil is an ideal model for studying the behavior of a scavenger unencumbered by the pressures of surviving within an extensive large predator or scavenger guild. Historically, a suite of large carnivores inhabiting mainland Australia may have driven the devil and its ancestors to exploit carrion as a dietary niche left relatively vacant (Owen & Pemberton, 2005). Today, the devil is restricted to Tasmania in the role of an apex scavenger, with no natural predators (Guiler, 1970) and facing little interspecific competition for resources save for the rare spotted‐tailed quoll (*Dasyurus maculatus*; Andersen et al., 2017b; Jones & Barmuta, 1998), the feral cat (*Felis catus*; Cunningham et al., 2020; Hollings et al., 2016), and an assortment of facultative scavenging bird species including the forest raven (*Corvus tasmanicus*; Cunningham et al., 2018) and wedge‐tailed eagle (*Aquila audax*; Olsen, 2005). This sets the species apart from other prominent mammalian scavengers such as the spotted hyena (*Crocuta crocuta*), which alters foraging activity in response to the density of larger predators and competitors (Pereira et al., 2013) and the wolverine (*Gulo gulo*), which benefits from their provision of carrion (Khalil et al., 2014; Mattisson et al., 2011). Like many scavengers, devils are considered to be generalist and opportunistic carnivores, consuming a wide range of food items (Andersen et al., 2017b; Bell et al., 2020; Guiler, 1970; Jones & Barmuta, 1998; Pemberton et al., 2008; Rogers, Fox, et al., 2016; Rogers, Fung, et al., 2016; Taylor, 1986). However, these studies also show evidence that individual devils can have divergent dietary choices and foraging strategies, arising from many proposed drivers, including age (Bell et al., 2020; Guiler, 1970; Jones & Barmuta, 1998), sex (Jones & Barmuta, 1998), seasonal changes (Jones & Barmuta, 1998), and interspecific competition (Andersen et al., 2017b; Jones & Barmuta, 1998). The primary method used to investigate devil diet choice—morphological scat analysis—is limited to providing data that represent only short periods (typically 1 day) and can misrepresent the proportions of certain food items due to differences in digestibility (Dickman & Huang, 1988; Lockie, 1959). Recently, stable isotope analysis has been employed to overcome these limitations (Bell et al., 2020), but wide‐scale analysis of the extent of dietary specialization in individual devils remains largely unquantified.

Stable isotope analysis has previously been employed to describe scavenger diets and their responses to external pressures (Lambertucci et al., 2018; Perrig et al., 2016; Tran, 2014), as well as to demonstrate individual diet specialization among seabirds (Anderson et al., 2008; Masello et al., 2013) and urban coyotes scavenging from anthropogenic sources (Larson et al., 2020). We sought to use bulk stable isotope analysis to measure the extent of dietary variation in devils over several weeks. As stable isotopes reflect the nutrients assimilated into body tissues (Crawford et al., 2008), rather than those that are excreted, they can provide a more reliable measure of dietary composition than scat samples alone. Where stable isotopes are locked in a keratin matrix following assimilation, predictable tissue growth can then be used to measure change in diet over extended periods of time (Rogers, Fox, et al., 2016; Rogers, Fung, et al., 2016). Carbon isotopic composition and fractionation varies greatly between the plant species forming the base of the food web, based on pathways of photosynthesis, characteristics of leaf gas exchange, and environmental effects such as water supply and the use of fertilizer (Bender et al., 1973; Cernusak et al., 2013; Farquhar et al., 1982; O’Leary, 1981). These differences are passed up through the food chain, providing an indication of the habitats that prey, predator, and scavenger species are feeding in. Nitrogen isotopic composition is also passed predictably from food item to consumer, allowing one to map an individual's trophic position and niche width (Bearhop et al., 2004; Layman et al., 2012). Using this technique, we aimed first to test our hypothesis that individual devils are capable of dietary specialization in both trophic level (detected via stable‐nitrogen isotope values) and feeding area (detected via stable‐carbon isotope values), despite feeding broadly as a population. Second, we tested whether the degree of specialization varied with inherent characteristics (sex, age, and size) or environmental effects (site and intraspecific competition level).

## 2 METHODS

### 2.1 Whisker sample collection

We sampled 71 individual devils (Table S1) between August 1 and October 4, 2018, captured overnight in custom‐made PVC pipe traps, across seven study sites in north‐western Tasmania (Figure S1). Sex, mass, and head width were recorded, and age was assessed based on the extent of canine eruption and other markers modified from Pemberton (1990). The longest posterior mystacial whisker (A–F, Figure S2) was collected by cutting as close to the skin as possible with scissors, and its position was recorded. Two study sites (Dip River and New Haven; Figure S1) were classified as having lowered intraspecific competition, following severe population decline in 2013 and 2014, respectively, after the introduction of Devil Facial Tumor Disease (DFTD; C. Hughes, unpublished data). DFTD causes an average local population decline of 77% in the 5 years following its introduction to a site (Lazenby et al., 2018). Therefore, it is reasonable to assume that the individuals inhabiting disease‐affected sites experience severely reduced competition levels compared with those in unaffected sites and are not at carrying capacity. All other sites were classified as having normal competition.

### 2.2 Whisker preparation

Each whisker was measured to the nearest 0.5 mm and an estimated intradermal length was added to calculate its estimated total length (M. Attard, unpublished data, Table S1). The amount of time each whisker represented was then modelled based on a discrete von Bertalanffy equation (von Bertalanffy, 1957; Hall‐Aspland et al., 2005; Rogers, Fox, et al., 2016; Rogers, Fung, et al., 2016) so that the whiskers could be sectioned into lengths representing a period of a few days. Whiskers were cleaned to remove lipids and other debris by washing once in ultrapure water and twice in a 2:1 chloroform:methanol solution for 20 min each. Three segments of each whisker weighing between 0.2 and 0.5 mg (mean = 0.32 ± 0.08 mg) were cut and placed in tin capsules for analysis. Each isotope segment (*n* = 213) represented approximately 2.7 ± 1.5 days of growth (and thus assimilated diet), falling between May 19 and September 1, 2018. A buffer section of approximately 11.3 ± 2.2 days of growth was also cut between each isotope segment, ensuring a greater degree of independence between samples. In total, the three analyzed segments represented approximately 1 month of isotope data.

### 2.3 Potential food item collection and preparation

Potential Tasmanian devil food items (Table 1) were opportunistically collected, usually as roadkill, from study sites and the rural areas surrounding Smithton, Irishtown, Roger River, and Montagu between 2016 and 2019 and frozen at −20°C. A feather from each bird was collected and soaked once in ultrapure water and twice in a 2:1 chloroform:methanol solution for 20 min each. Two samples of the rachis weighing between 0.2 and 0.5 mg were cut and placed into tin capsules for analysis. A sample of muscle tissue (usually from the thigh or torso) was cut from each mammal and snake, rinsed thoroughly twice in ultrapure water, and left to air dry. The outer edges of the muscle sample were then cut using a scalpel to remove as much potentially contaminated tissue as possible. Invertebrates were cleaned by rinsing twice thoroughly in ultrapure water and analyzed whole. Mammal, snake, and invertebrate samples were then placed in a freeze‐dryer (Alpha 1‐4 LSCbasic, CHRIST) for at least 12 h and ground into a powder using an oscillating mill (MM 200, Retsch). Two samples of powder weighing between 0.2 and 0.5 mg from each individual were taken and placed into tin capsules for analysis.

**TABLE 1:** Mean δ^15^N and δ^13^C values (‰) for potential Tasmanian devil food items ± SE

Species	*n*	Mean δ^15^N (‰)	Mean δ^13^C (‰)
Mammals			
Brushtail possum (*Trichosurus vulpecula*)	2	5.4 ± 0.8	−25.5 ± 0.6
European hare (*Lepus europaeus*)	2	6.1 ± 1.0	−27.7 ± 0.8
Red‐necked wallaby (*Macropus rufogriseus*)	3	3.3 ± 0.5	−28.1 ± 0.4
Southern brown bandicoot (*Isoodon obesulus*)	3	7.0 ± 1.5	−25.8 ± 0.3
Spotted‐tailed quoll (*Dasyurus maculatus*)	6	8.2 ± 0.6	−25.6 ± 0.4
Tasmanian pademelon (*Thylogale billardierii*)	5	4.9 ± 0.7	−28.0 ± 0.5
Birds			
Australasian swamphen (*Porphyrio melanotus*)	2	8.0 ± 0.1	−28.3 ± 2.7
Black currawong (*Strepera fuliginosa*)	2	6.2 ± 0.4	−23.2 ± 0.3
Forest raven (*Corvus tasmanicus*)	4	8.7 ± 0.6	−24.4 ± 1.5
Green rosella (*Platycercus caledonicus*)	4	−0.6 ± 1.5	−24.6 ± 0.9
Laughing kookaburra (*Dacelo novaeguineae*)	2	7.3 ± 1.7	−24.3 ± 0.1
Masked lapwing (*Vanellus miles*)	2	7.3 ± 0.2	−25.5 ± 0.5
Swamp harrier (*Circus approximans*)	3	10.4 ± 1.6	−16.4 ± 3.5
Tasmanian nativehen (*Tribonyx mortierii*)	4	8.0 ± 0.8	−27.3 ± 0.8
Reptiles			
Tiger snake (*Notechis scutatus*)	3	7.9 ± 0.6	−25.6 ± 1.2
Invertebrates			
Carrion beetle (*Ptomaphila lacrymosa*)	4	9.5 ± 1.6	−26.2 ± 0.6
Round fungus beetle (*Pseudonemadus* sp.)	8	9.4 ± 0.5	−25.8 ± 0.2
Rove beetle (*Creophilus lanio*)	2	11.9 ± 1.2	−28.1 ± 0.3

### 2.4 Stable isotope analysis

All whisker and food item samples were combusted in an elemental analyzer (Flash 2000 Organic Elemental Analyser, Thermo Scientific) and the nitrogen and carbon isotope ratios (δ^15^N and δ^13^C) were determined using a continuous flow isotope ratio mass spectrometer (Delta V Advantage, Thermo Scientific) at the Bioanalytical Mass Spectrometry Facility, University of New South Wales, Australia. Isotope ratios are expressed using standard delta notation as parts per thousand (‰) and corrected to atmospheric N_2_ (Air) for δ^15^N and Vienna Pee Dee Belemnite (VPDB) for δ^13^C values (Bond & Hobson, 2012). Instrument drift and measurement error were corrected using international standards USGS40 (δ^15^N_AIR_ = −4.52 ± 0.06‰; δ^13^C_VPDB‐LSVEC_ = −26.39 ± 0.04‰) and USGS41a (δ^15^N_AIR_ = 47.55 ± 0.15‰; δ^13^C_VPDB‐LSVEC_ =36.55 ± 0.08‰; Qi et al., 2003, 2016).

### 2.5 Mapping the Tasmanian isoscape

In order to confirm that nitrogen and carbon isotopes could be used to indicate devil trophic position and foraging location, we plotted their mean δ^15^N and δ^13^C values against those of potential food items. Discrimination factors from Newsome et al. (2010) were used to account for trophic enrichment.

### 2.6 Specialization

Nitrogen and carbon specialization indices (NSI and CSI) were calculated for each devil to describe the variance in δ^15^N and δ^13^C values over a 1‐month period (Roughgarden, 1972). The degree of specialization was calculated using the equation:

$$\text{SI}=\frac{\text{INW}}{\left(\text{INW}+\text{BINW}\right)}$$

where SI is the specialization index; INW is the individual niche width, the variance of isotopic values along the whisker; and BINW is the between‐individual niche width, the total variance of isotopic values within the sampled population. BINW was calculated for the total population across all seven study sites as these sites are not discrete, with individuals frequently traveling between neighboring sites (C. Hughes, unpublished data). Individuals that occupied over 50% of the total niche width (TNW = INW + BINW) were classified as nitrogen or carbon generalists (SI > 0.5), while those restricted to less than 20% of the total niche width were classified as specialists (SI < 0.2), based upon conventions introduced in the southern elephant seal (Hückstädt et al., 2011). Individuals with a specialization index between 0.2 and 0.5 were classified as intermediates.

### 2.7 Model development

We tested the effects of five variables (age, sex, size, intraspecific competition level, and site) on NSI and CSI. Age, sex, and size were chosen as individual characteristics due to their previously recorded effects on diet content in morphological scat analysis and δ^15^N values (Bell et al., 2020; Guiler, 1970; Jones & Barmuta, 1998). Age was restricted to a binary effect, with devils aged 2–4 years grouped as “adults,” while still‐maturing 1‐year olds were defined as “yearlings.” Old devils (over 4 years) and juveniles (under 1 year) were excluded as they were trapped in low numbers during the collection period. Both mass and head width were initially included as indicators of the size of the individual, though only mass was retained due to high correlation between these two variables.

Intraspecific competition level and site were chosen as potential environmental effects on NSI and CSI. Competition has been shown to drive both increased and decreased specialization across taxa (Araújo et al., 2011; Bolnick et al., 2003). While interspecific competition is reduced for the devil compared with other scavengers, individuals still fiercely compete for resources within their overlapping home ranges (Guiler, 1970; Pemberton & Renouf, 1993). Site was included in order to determine whether differences in specialization were due simply to access to different resources.

### 2.8 Statistical analysis

All statistical analyses were conducted in RStudio (R Core Team, 2020). Linear models were fitted with NSI and CSI as response variables and including the fixed effects of age, sex, mass, intraspecific competition level, and site. Top models were generated and selected using the R package MuMIn (Bartoń, 2020) based on changes in the Akaike information criterion corrected for sample size (AIC_c_), with ΔAIC_c_ < 2 indicating substantial support (Burnham & Anderson, 2002). These criteria are consistent with previous studies on Tasmanian devil diet (Bell et al., 2020), enabling easy comparison. Top models were checked for the assumption of normality using the Eco‐Stats package (Warton, 2020) to ensure that all data were contained within 95% of quantile plot envelopes. CSI did not meet this assumption and was therefore log‐transformed. Model generation and selection were repeated using log(CSI) as the response variable. ANOVAs were then performed to compare the fit of top ranked models with the null model. An ANOVA was also later applied to NSI and log(CSI) values to determine whether those classified as nitrogen specialists were also likely to be classified as carbon specialists.

## 3 RESULTS

### 3.1 Tasmanian isoscape

**FIGURE 2:**
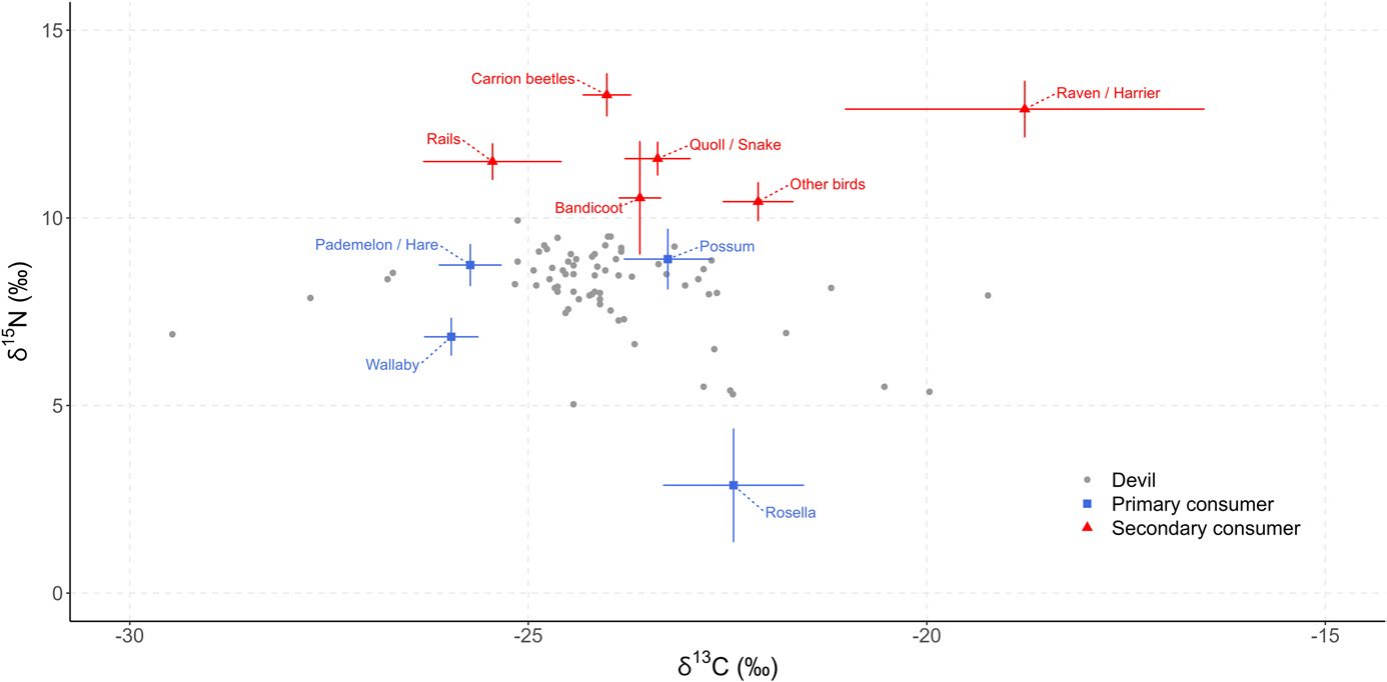
Mean δ^15^N and δ^13^C values (‰) for individual Tasmanian devils (*n* = 71) and potential food items (±SE) after the addition of trophic discrimination factors (+3.5‰ for nitrogen; +2.2‰ for carbon (Newsome et al., 2010)). Where isotopic values were very similar, species were combined to form one of ten food item groups: carrion beetles (*P*. *lacrymosa*, *Pseudonemadus* sp., *C. lanio*); raven/harrier (*C*. *tasmanicus*, *C. approximans*); rails (*P*. *melanotus*, *T. mortierii*); quoll/snake (*D*. *maculatus*, *N. scutatus*); bandicoot (*I. obesulus*); other birds (*S*. *fuliginosa*, *D*. *novaeguineae*, *V. miles*); pademelon/hare (*T*. *billardierii*, *L. europaeus*); possum (*T. vulpecula*); wallaby (*M. rufogriseus*); and rosella (*P. caledonicus*)

Mean isotopic values for other Tasmanian species (Table 1) ranged from −0.6 to 11.9‰ for δ^15^N and −28.3 to −16.4‰ for δ^13^C (Figure 2).

### 3.2 Nitrogen and carbon specialization

**FIGURE 3:**
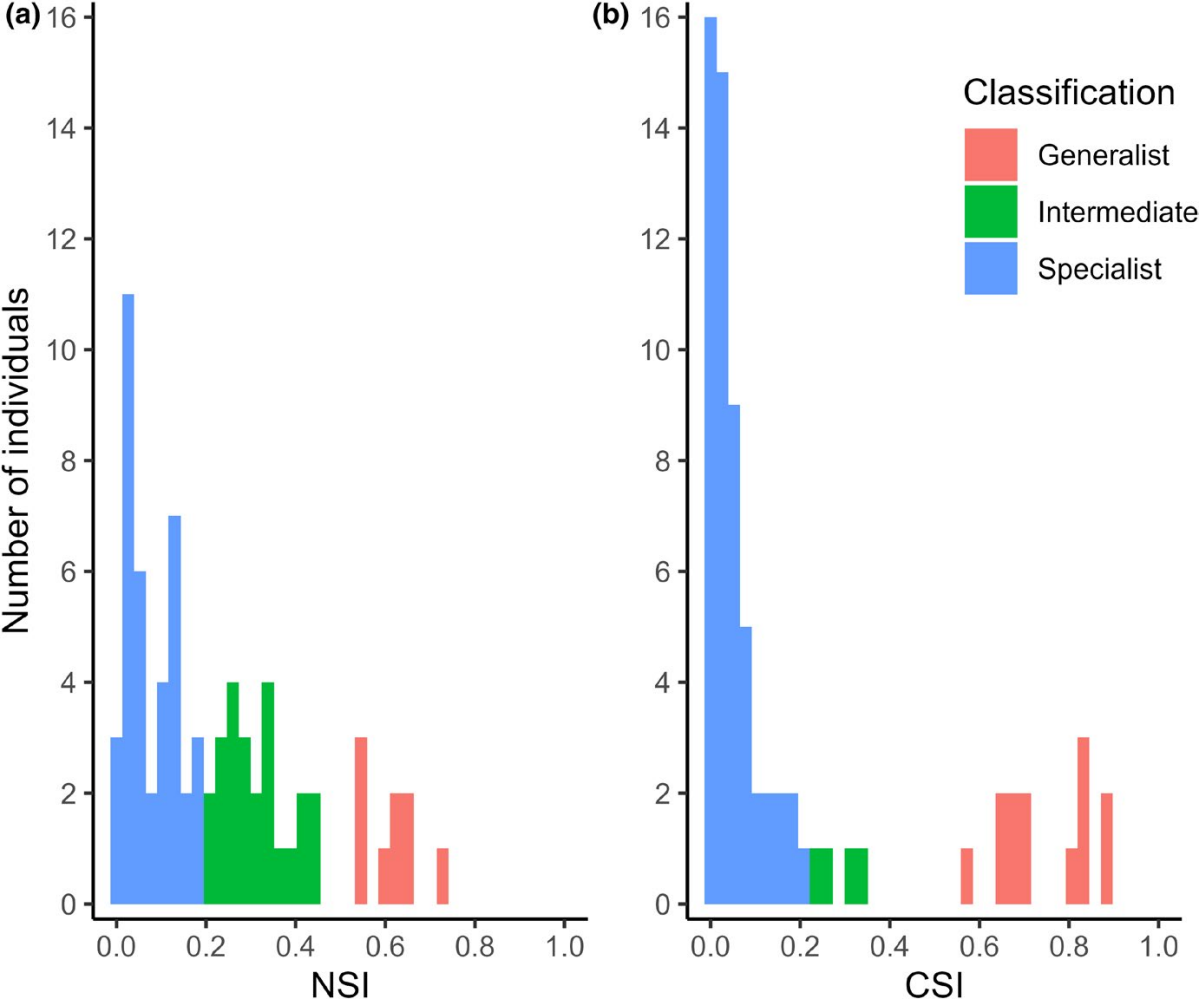
Frequency distribution of the specialization index (INW/TNW) of Tasmanian devils (*n* = 71): (a) For δ^15^N values (NSI), where 53.5% of individuals are classified as; (b) For δ^13^C values (CSI) where 76.1% individuals are classified as specialists

As a population, δ^15^N values ranged from 4.1‰ to 10.8‰, while δ^13^C values ranged from −30.7‰ to −14.3‰. Only nine individuals (12.7%) had an NSI of over 0.5, classifying them as nitrogen generalists; most devils were classified as nitrogen specialists (*n* = 38, 53.5%), with an NSI of under 0.2 (Figure 3a). Only 13 devils (18.3%) had a CSI over 0.5, classifying them as carbon generalists; the majority (*n* = 54, 76.1%) had a CSI under 0.2 and were classified as carbon specialists (Figure 3b).

### 3.3 Drivers of diet specialization

**FIGURE 4:**
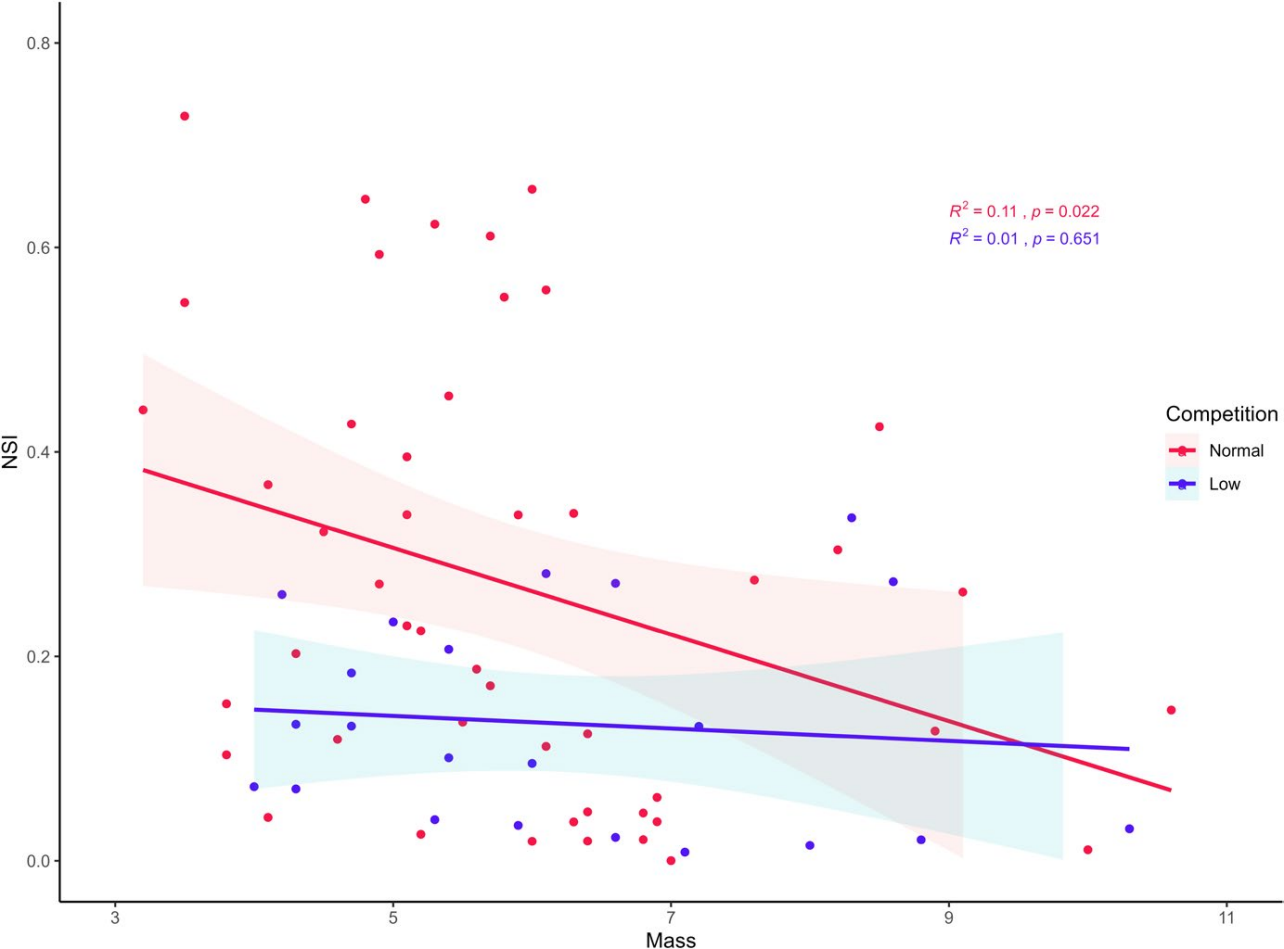
There is a negative relationship between nitrogen specialization index (NSI) and mass (kg) of Tasmanian devils in addition to an effect of intraspecific competition level (linear model: *n* = 70, *p* < .01, *R* ^2^ = .17). Larger devils were more likely to be classified as nitrogen specialists, particularly in densely populated areas with greater intraspecific competition. Nitrogen generalists (NSI > 0.5) were always small devils (≤6.1 kg) in competitive populations. No interactive effect of mass and intraspecific competition was found

Mass and intraspecific competition level were retained in all top models explaining variation in NSI (*n* = 70 following removal of missing data, Table 2). We found a negative relationship between NSI and mass as well as an effect of competition level, indicating that larger individuals were more likely to be classified as nitrogen specialists, particularly at competitive sites (*p* < .01, Figure 4). The second ranked model retained the interaction effect of mass and competition level and the third retained sex, but neither of these variables explained any additional variation of NSI.

**TABLE 2:** Summary of linear models for NSI (specialization index of δ^15^N values (‰)) where ΔAIC_c_ < 2

Model rank	Intercept	Competition	Mass	Sex	Competition*Mass	*df*	logLik	ΔAIC_c_	Weight	*p*
1	0.323	+	−0.030			4	22.234	0.00	0.447	.0019
2	0.173	+	−0.006		+	5	23.141	0.51	0.347	.0027
3	0.352	+	−0.038	+		5	22.623	1.55	0.206	.0042

Note

Age, sex, mass, intraspecific competition level, and site were included as fixed variables. *p* values are compared with the null model.

The top ranked model for CSI was the null, indicating that CSI did not change in response to any of the factors included in our model (mass, sex, age, intraspecific competition, or site). Additionally, we found no relationship between carbon and nitrogen specialization, that is, those classified as nitrogen specialists were not more likely than nitrogen generalists to also be classified as carbon specialists.

## 4 DISCUSSION

We show that the Tasmanian devil can be an individual dietary specialist while feeding on a wide range of food items as a population. Indeed, very few individuals were classified as true generalists, most feeding within a narrow trophic niche (inferred from stable‐nitrogen isotope values) and within a relatively restricted area (inferred from stable‐carbon isotope values). Therefore, we suggest that devils feed much less opportunistically than previously assumed.

Heavier devils were more likely to be trophic specialists, regardless of sex or age. This could indicate that mass is an important driver of diet choice or, alternatively, that dietary specialization is a more efficient foraging strategy, allowing individuals to attain a greater mass. Greater body size of Andean condors (*Vultur gryphus*) is associated with more restricted foraging schedules concentrated in the morning, when wind resources and availability of carcasses were optimal (Alarcón et al., 2017). Similarly, other large scavengers may be able to feed on a desired resource more easily, whether by being better able to defend carcasses, by consuming them more quickly (Ruxton & Houston, 2004), by moving through the landscape more efficiently, or by supplementing their diet by hunting larger prey items (Carbone et al., 2007; Tucker et al., 2014, 2016). Though hunting and killing events by wild devils have recently been captured on video (Andersen et al., 2020, C. Hughes, unpublished data), data are still limited and it is yet unknown what influence body size may have on both hunting frequency and prey choice.

Devils inhabiting sites with lower levels of intraspecific competition were more likely to feed within a narrow trophic niche. This is perhaps unsurprising, as carcass defense would be easier at sparsely populated sites, where direct contact between individuals is less frequent (Hamede et al., 2008). Although mammalian scavengers are expected to feed opportunistically, our results show that the devil, an animal that both heavily relies on and is highly adapted to scavenging, adopts a more specialist feeding strategy particularly in response to reduced competition, as do predatory taxa (Araújo et al., 2011; Bolnick et al., 2003, and references within, Larson et al., 2020). High interspecific competition within scavenger guilds increases the importance of efficiency in carcass detection and consumption (Sebastián‐González et al., 2013) and likely favors individuals reducing their time spent seeking out a specific food source. Devils are perhaps unique among scavenging mammals in the great extent to which they can specialize because they have been released from the pressures of any large, abundant competitors following the extinction of the thylacine in the last century (Owen, 2003). Further investigation is warranted into how devil diet has responded to diverse levels of interspecific competition throughout history. However, caution should be exercised when interpreting dietary specialization as conscious choice made by the individual. Medium‐sized mammals such as the Tasmanian pademelon (*Thylogale billardierii*), the red‐necked wallaby (*Macropus rufogriseus*), and the brushtail possum (*Trichosurus vulpecula*) are common targets for hunting or vehicular strike in Tasmania (Animal Welfare Advisory Committee Tasmania, 2003; Hobday & Minstrell, 2008). Thus, it is likely that higher levels of specialization within non‐competitive populations of devils are the result of an overabundance of these particular resources.

The prevalence of specialization of carbon isotopic composition provides evidence that devils are systematic in their foraging. The majority of individuals in this study were classified as extreme specialists for carbon isotopic values, suggesting they foraged in specific areas, regardless of trophic niche breadth and despite our field sites covering a wide range of Tasmanian habitat types. Devils are known to preferentially travel along roads and ecotones that facilitate rapid movement through their home ranges (Andersen et al., 2017a; Guiler, 1970). It is likely that these habitat features are also favored feeding grounds, particularly if roads provide more carcasses as a result of vehicle strike. Roadside and anthropogenically disturbed vegetation may vary in carbon isotope composition compared with undisturbed vegetation in response to differences in water and nutrient availability (Condon et al., 1992; Stewart et al., 1995) or to increased exposure to sunlight and pollutants (Battipaglia et al., 2009). A narrow carbon isotopic range may simply indicate that it is prey species that are feeding in specific areas, rather than the devils themselves. However, there is considerable overlap in the movements of devils and herbivore species such as pademelons and wallabies, that also preference ecotones between forest and pasture (Johnson, 1980; Wahungu et al., 2001; While & McArthur, 2005). Thus, it is likely that both devils and their prey are feeding in similar areas. Measuring the carbon isotopic signatures of vegetation within a Tasmanian context could therefore be used to identify important devil feeding sites.

Although devils have shown differences in diet based on sex and age (Jones & Barmuta, 1998), particularly in winter, we did not find that this translated into a difference in the extent of their dietary specialization. The broader isotopic niches seen in juvenile devils, suggested to reflect the weaning period, have likely already become restricted as animals approach sexual maturity between 1 and 2 years old (Bell et al., 2020), the age of our youngest individuals. As males and older individuals are typically larger than females and young adults, it is possible that dietary partitioning by sex or age is linked with a relationship between body mass and trophic feeding level that should be further explored. If larger individuals are specializing on the same resources, this could be used to identify which food items are considered most desirable to devils when their choices are uninhibited by intraspecific competition. This study considered specialization only over 1 month in winter, the season in which devils show lower specialization in diet as a population (Jones & Barmuta, 1998). Though this focus allowed us to compare individuals over the same time period, it also limited our ability to model the relationship between isotope composition and the degree of specialization. Differences in dietary specialization may become even more pronounced in other seasons as resource availability and population dynamics change. Measuring changes in stable isotope ratios over a year or even an individual's entire 6‐year lifespan would allow us to assess whether individuals who feed at a particular trophic level are more likely to be specialists. To analyze whether feeding specialization varies in response to changes in resource availability and intraspecific competition, one could also measure the difference in stable isotope ratios between habitats and before and after DFTD‐induced population decline.

The stable‐nitrogen isotope values of potential devil food items that we observed indicate they can be used to plot the trophic position of terrestrial Tasmanian species. Our results suggest that the Tasmanian devil primarily feeds on medium‐sized mammals such as Tasmanian pademelons, red‐necked wallabies, and brushtail possums, as observed in previous studies that used morphological scat analysis (Andersen et al., 2017b; Guiler, 1970; Jones & Barmuta, 1998; Pemberton et al., 2008; Rogers, Fox, et al., 2016; Rogers, Fung, et al., 2016; Taylor, 1986). It has been theorized that higher stable‐nitrogen isotope values in devils may indicate a greater reliance on birds over herbivorous mammals (Bell et al., 2020), though our results show that very low values may also be evidence of feeding on rosellas and other herbivorous birds. This study did not include a detailed analysis of diet using Bayesian mixing models, which is an essential step if we are to interpret more accurately what particular stable‐nitrogen and stable‐carbon isotope ratios mean in terms of diet composition. However, our results could be used as a foundation for choosing which potential food groups to include.

In recent years, the Tasmanian devil has suffered from the outbreak of DFTD, which has reduced local populations by an average of 77% across the disease range (Lazenby et al., 2018). At these low densities, other threats to their survival, such as road traffic and land clearing, become even more significant (McCallum & Jones, 2006). We have shown that devils are capable of feeding generally where competition is higher and resources are likely more restricted, indicating their potential to adapt to the rapidly changing environment surrounding them. This is particularly important for species that rely on scavenging, as opposed to those that scavenge to supplement their diets (Ruxton & Houston, 2004). However, most individuals use a narrow dietary niche, and it is unclear whether their foraging strategies are flexible enough to change quickly or if they are relatively fixed from early development. Thus, the degree to which devils are able to broaden their diets may play a role in the success of their rehabilitation in the wake of disease and population decline.

## CONFLICT OF INTEREST

The authors have no conflicts of interest to declare.

## AUTHOR CONTRIBUTIONS

**Anna C. Lewis:** Conceptualization (equal); Data curation (equal); Formal analysis (equal); Investigation (equal); Methodology (equal); Project administration (equal); Visualization (equal); Writing‐original draft (equal); Writing‐review & editing (equal). **Channing Hughes:** Funding acquisition (equal); Investigation (equal); Project administration (equal); Resources (equal); Supervision (equal); Writing‐review & editing (equal). **Tracey Leeanne Rogers:** Conceptualization (equal); Formal analysis (equal); Funding acquisition (equal); Investigation (equal); Methodology (equal); Project administration (equal); Resources (equal); Supervision (equal); Writing‐review & editing (equal).

## Supporting information

Supinfo S1

## Data Availability

Data are available from the Dryad Digital Repository https://doi.org/10.5061/dryad.vhhmgqnvh.
