# Supplementary material for: Effects of intraspecific competition and body mass on diet specialization in a mammalian scavenger
Source: Ecol Evol. 2022 Jan 11;12(1):e8338. doi: 10.1002/ece3.8338 (PMC8794717; doi:10.1002/ece3.8338)
Supplement: Supplementary file 1 — Supinfo S1 [file ECE3-12-e8338-s001.docx]

**Supplementary Table 1.** Data for individual Tasmanian devils (n = 71) included in this study. Whisker position illustrated in Supplementary Figure 2. Estimated intradermal length of whisker based on M. Attard, unpublished data. NSI is nitrogen specialisation index and CSI is carbon specialisation index, where values < 0.2 are classified as specialist and those > 0.5 are classified as generalist.

| Sampling date | Site | Competition | Animal ID | Sex | Age group | Mass (kg) | Whisker position | Estimated intradermal whisker length (mm) | Estimated total whisker length (mm) | NSI | CSI |
| --- | --- | --- | --- | --- | --- | --- | --- | --- | --- | --- | --- |
| 24/08/18 | Milkshake Hills | Normal | Zagreb | Male | Adult | 8.5 | D | 5.7 | 83.7 | 0.42 | 0.08 |
| 10/09/18 | Wedge Plains | Normal | Frangipani | Female | Adult | 5.9 | E | 5.5 | 102.0 | 0.34 | 0.66 |
| 5/09/18 | Blackwater | Normal | Furina | Female | Adult | 6.4 | C | 5.4 | 86.4 | 0.05 | 0.01 |
| 3/09/18 | Blackwater | Normal | Odile | Female | Adult | 5.8 | C | 5.4 | 126.4 | 0.55 | 0.01 |
| 21/08/18 | Milkshake Hills | Normal | Dodoma | Female | Adult | 5.5 | F | 5.5 | 106.0 | 0.14 | 0.00 |
| 4/10/18 | Dip River | Low | Mimosa | Female | Adult | 5.4 | C | 5.4 | 118.9 | 0.21 | 0.07 |
| 15/09/18 | Sumac Road | Normal | Arno | Male | Adult | 6.0 | D | 5.7 | 103.7 | 0.66 | 0.84 |
| 24/09/18 | Wuthering Heights | Normal | Furud | Male | Adult |  | E | 5.5 | 94.0 | 0.05 | 0.02 |
| 21/09/18 | Wuthering Heights | Normal | Jysetha | Female | Adult | 6.1 | E | 5.5 | 90.5 | 0.11 | 0.03 |
| 23/09/18 | Wuthering Heights | Normal | Arcturus | Male | Adult | 6.8 | E | 5.5 | 91.0 | 0.02 | 0.02 |
| 31/08/18 | Blackwater | Normal | Purcell | Male | Adult | 10.6 | F | 5.5 | 94.0 | 0.15 | 0.02 |
| 23/09/18 | Wuthering Heights | Normal | Deucalion | Male | Adult | 7.6 | E | 5.5 | 113.0 | 0.27 | 0.04 |
| 21/08/18 | Milkshake Hills | Normal | Clyde | Male | Adult | 9.1 | B | 4.9 | 77.4 | 0.26 | 0.04 |
| 23/09/18 | Wuthering Heights | Normal | Alcott | Female | Adult | 4.9 | D | 5.7 | 132.2 | 0.27 | 0.01 |
| 2/09/18 | Blackwater | Normal | Arcanine | Male | Adult | 5.1 | C | 5.4 | 123.4 | 0.40 | 0.03 |
| 14/09/18 | Wedge Plains | Normal | Haloumi | Male | Adult | 8.9 | E | 5.5 | 88.0 | 0.13 | 0.02 |
| 11/08/18 | New Haven | Low | Pontchartrain | Male | Adult | 10.3 | C | 5.4 | 115.4 | 0.03 | 0.65 |
| 21/09/18 | Wuthering Heights | Normal | Denebola | Female | Yearling | 4.8 | F | 5.5 | 87.0 | 0.65 | 0.01 |
| 23/09/18 | Wuthering Heights | Normal | Quantz | Male | Yearling | 6.3 | C | 5.4 | 127.4 | 0.34 | 0.01 |
| 13/08/18 | New Haven | Low | Bogoria | Female | Adult | 6.0 | D | 5.7 | 113.7 | 0.10 | 0.03 |
| 12/08/18 | New Haven | Low | Naivasha | Female | Adult | 5.4 | B | 4.9 | 95.4 | 0.10 | 0.11 |
| 9/08/18 | New Haven | Low | Rudolf | Male | Adult | 8.6 | C | 5.4 | 151.4 | 0.27 | 0.34 |
| 2/09/18 | Blackwater | Normal | Yaroslavna | Female | Adult | 5.3 | F | 5.5 | 73.0 | 0.62 | 0.26 |
| 29/09/18 | Dip River | Low | Verbena | Female | Adult | 4.3 | D | 5.7 | 97.2 | 0.07 | 0.00 |
| 11/09/18 | Wedge Plains | Normal | Alverca | Female | Adult | 6.9 | C | 5.4 | 111.4 | 0.04 | 0.05 |
| 18/09/18 | Sumac Road | Normal | Tanana | Female | Adult | 5.7 | D | 5.7 | 146.7 | 0.17 | 0.30 |
| 18/09/18 | Sumac Road | Normal | Ranelva | Female | Adult | 5.7 | E | 5.5 | 119.0 | 0.61 | 0.04 |
| 16/09/18 | Sumac Road | Normal | Liffey | Male | Adult | 6.3 | C | 5.4 | 140.4 | 0.04 | 0.02 |
| 15/09/18 | Wedge Plains | Normal | Kashkaval | Male | Adult | 8.2 | D | 5.7 | 110.7 | 0.30 | 0.07 |
| 22/09/18 | Wuthering Heights | Normal | Wei | Female | Adult | 5.6 | D | 5.7 | 144.2 | 0.19 | 0.03 |
| 22/09/18 | Wuthering Heights | Normal | Xerouba | Female | Adult | 6.8 | D | 5.7 | 126.2 | 0.05 | 0.83 |
| 16/09/18 | Sumac Road | Normal | Biafra | Female | Yearling | 3.5 | C | 5.4 | 128.9 | 0.55 | 0.00 |
| 3/10/18 | Dip River | Low | Sorrel | Male | Yearling | 6.6 | C | 5.4 | 147.9 | 0.02 | 0.05 |
| 10/08/18 | New Haven | Low | Alonnah | Male | Adult | 8.0 | D | 5.7 | 86.7 | 0.02 | 0.02 |
| 8/08/18 | New Haven | Low | Yakus | Male | Yearling | 6.6 | D | 5.7 | 112.7 | 0.27 | 0.71 |
| 9/08/18 | New Haven | Low | Queraba | Female | Adult | 7.2 | C | 5.4 | 78.4 | 0.13 | 0.82 |
| 1/09/18 | Blackwater | Normal | Bellsprout | Female | Yearling | 4.3 | F | 5.5 | 71.0 | 0.20 | 0.64 |
| 12/08/18 | New Haven | Low | Zug | Male | Yearling | 7.1 | F | 5.5 | 79.5 | 0.01 | 0.88 |
| 2/10/18 | Dip River | Low | Casuarina | Female | Adult | 4.0 | E | 5.5 | 130.0 | 0.07 | 0.56 |
| 2/10/18 | Dip River | Low | Wattle | Male | Yearling | 8.3 | F | 5.5 | 82.5 | 0.34 | 0.89 |
| 13/09/18 | Wedge Plains | Normal | Imbe | Male | Yearling | 6.9 | D | 5.7 | 144.2 | 0.06 | 0.16 |
| 11/08/18 | New Haven | Low | Hellyer | Male | Adult | 8.8 | D | 5.7 | 110.2 | 0.02 | 0.01 |
| 8/08/18 | New Haven | Low | Volta | Female | Yearling | 4.7 | F | 5.5 | 74.5 | 0.18 | 0.00 |
| 8/08/18 | New Haven | Low | Urmia | Female | Yearling | 5.0 | F | 5.5 | 62.0 | 0.23 | 0.01 |
| 8/08/18 | New Haven | Low | Dunalley | Male | Yearling | 5.9 | C | 5.4 | 136.4 | 0.03 | 0.18 |
| 1/09/18 | Blackwater | Normal | Hitmonlee | Male | Yearling | 4.9 | C | 5.4 | 113.9 | 0.59 | 0.19 |
| 3/09/18 | Blackwater | Normal | Jirachi | Male | Yearling | 4.1 | C | 5.4 | 143.9 | 0.37 | 0.68 |
| 4/09/18 | Blackwater | Normal | Jigglypuff | Female | Yearling | 4.6 | F | 5.5 | 70.5 | 0.12 | 0.82 |
| 1/09/18 | Blackwater | Normal | Emolga | Female | Yearling | 3.2 | C | 5.4 | 123.4 | 0.44 | 0.24 |
| 2/09/18 | Blackwater | Normal | Incineroar | Male | Yearling | 6.0 | D | 5.7 | 142.7 | 0.02 | 0.01 |
| 24/09/18 | Wuthering Heights | Normal | Wisibada | Female | Yearling | 5.2 | D | 5.7 | 112.7 | 0.23 | 0.07 |
| 4/08/18 | Wuthering Heights | Normal | Narvi | Male | Yearling | 6.4 | C | 5.4 | 135.4 | 0.02 | 0.01 |
| 25/09/18 | Wuthering Heights | Normal | Deimos | Male | Yearling | 5.4 | C | 5.4 | 127.4 | 0.45 | 0.14 |
| 12/09/18 | Wedge Plains | Normal | Ubriaco | Male | Yearling | 5.1 | E | 5.5 | 102.0 | 0.34 | 0.06 |
| 11/09/18 | Wedge Plains | Normal | Ricotta | Female | Yearling | 4.5 | C | 5.4 | 127.4 | 0.32 | 0.01 |
| 21/09/18 | Wuthering Heights | Normal | Eridanus | Male | Yearling | 6.1 | E | 5.5 | 111.0 | 0.56 | 0.10 |
| 15/09/18 | Sumac Road | Normal | Tiber | Male | Yearling | 4.7 | D | 5.7 | 156.2 | 0.43 | 0.05 |
| 19/09/18 | Sumac Road | Normal | Faxa | Female | Yearling | 3.5 | B | 4.9 | 135.9 | 0.73 | 0.14 |
| 11/09/18 | Wedge Plains | Normal | Apricot | Female | Yearling | 5.2 | F | 5.5 | 70.0 | 0.03 | 0.00 |
| 18/08/18 | Milkshake Hills | Normal | Irazu | Male | Yearling | 4.1 | D | 5.7 | 140.2 | 0.04 | 0.06 |
| 12/08/18 | New Haven | Low | Yalka | Female | Yearling | 4.3 | D | 5.7 | 66.7 | 0.13 | 0.00 |
| 19/08/18 | Milkshake Hills | Normal | Chacaltaya | Female | Yearling | 3.8 | D | 5.7 | 88.7 | 0.15 | 0.70 |
| 25/09/18 | Wuthering Heights | Normal | Bergelmir | Male | Yearling | 6.4 | E | 5.5 | 121.5 | 0.12 | 0.02 |
| 29/09/18 | Dip River | Low | Nutmeg | Female | Yearling | 4.2 | C | 5.4 | 114.4 | 0.26 | 0.20 |
| 29/09/18 | Dip River | Low | Tipu | Male | Yearling | 6.1 | E | 5.5 | 128.0 | 0.28 | 0.04 |
| 16/09/18 | Sumac Road | Normal | Willamette | Male | Yearling | 7.0 | D | 5.7 | 103.2 | 0.00 | 0.04 |
| 1/08/18 | Wuthering Heights | Normal | Vindemiatrix | Female | Yearling | 3.8 | C | 5.4 | 127.9 | 0.10 | 0.02 |
| 9/08/18 | New Haven | Low | Wanaka | Female | Yearling | 5.3 | B | 4.9 | 138.4 | 0.04 | 0.06 |
| 1/08/18 | Wuthering Heights | Normal | Urna | Female | Yearling | 5.1 | C | 5.4 | 115.9 | 0.23 | 0.07 |
| 9/08/18 | New Haven | Low | Xavantes | Female | Yearling | 4.7 | C | 5.4 | 149.9 | 0.13 | 0.01 |
| 1/08/18 | Wuthering Heights | Normal | Jacrum | Male | Adult | 10.0 | B | 4.9 | 90.4 | 0.01 | 0.13 |


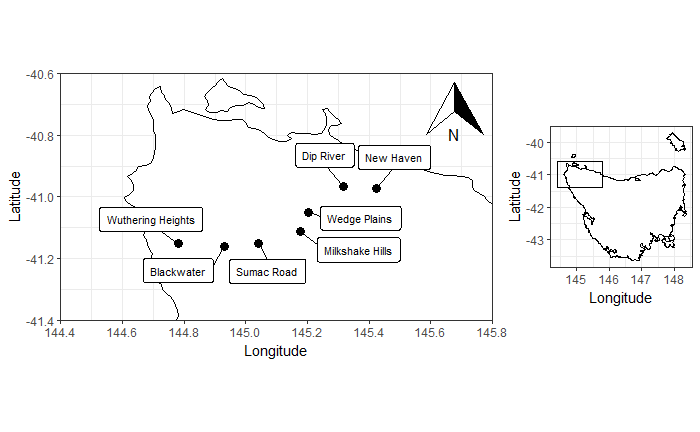


**Supplementary Figure 1.** Map of study area in north-west Tasmania, Australia.


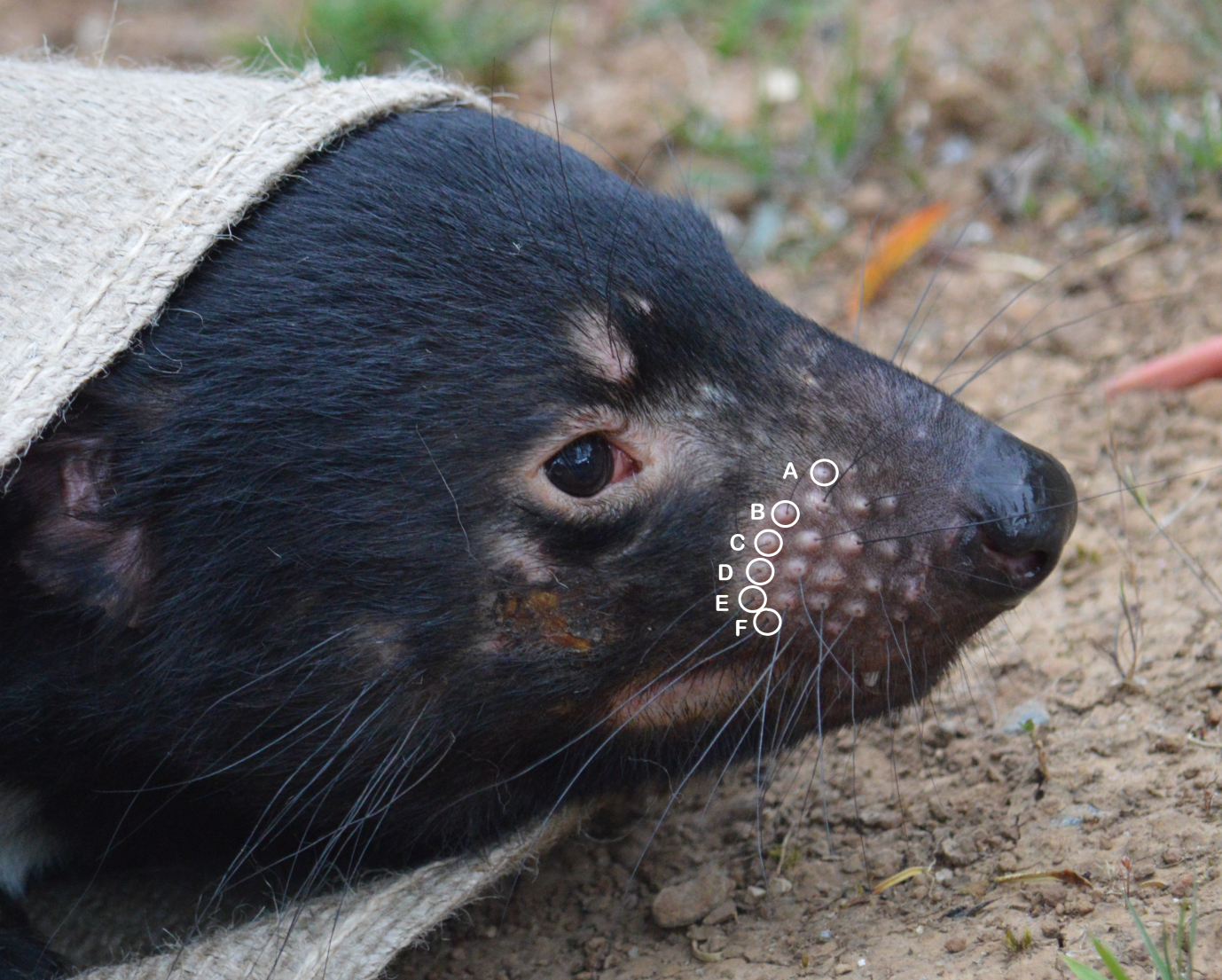


**Supplementary Figure 2.** Labelled whisker positions of the Tasmanian devil (photo by M. Pethybridge). The longest whisker from one of positions A-F was sampled.
